# Mid-gestation low-dose LPS administration results in female-specific excessive weight gain upon a western style diet in mouse offspring

**DOI:** 10.1038/s41598-020-76501-8

**Published:** 2020-11-12

**Authors:** Dorieke J. Dijkstra, Rikst Nynke Verkaik-Schakel, Sharon Eskandar, Alice Limonciel, Violeta Stojanovska, Sicco A. Scherjon, Torsten Plösch

**Affiliations:** 1 Department of Obstetrics and Gynaecology, University of Groningen, University Medical Center Groningen, Hanzeplein 1, CB22, 9713GZ Groningen, The Netherlands; 2 Section Molecular Neurobiology, Department of Biomedical Sciences of Cells and Systems, University of Groningen, University Medical Center Groningen, Groningen, The Netherlands; 3 Biocrates Life Sciences AG, Innsbruck, Austria; 4 Department of Environmental Immunology, Helmholtz Centre for Environmental Research, Leipzig, Germany; 5 Perinatal Neurobiology, Department of Human Medicine, School of Medicine and Health Sciences Carl von Ossietzky University Oldenburg, Oldenburg, Germany

**Keywords:** Feeding behaviour, Metabolic diseases, Reproductive biology, Experimental models of disease, Preclinical research

## Abstract

Gestational complications, including preeclampsia and gestational diabetes, have long-term adverse consequences for offspring’s metabolic and cardiovascular health. A low-grade systemic inflammatory response is likely mediating this. Here, we examine the consequences of LPS-induced gestational inflammation on offspring’s health in adulthood. LPS was administered to pregnant C57Bl/6J mice on gestational day 10.5. Maternal plasma metabolomics showed oxidative stress, remaining for at least 5 days after LPS administration, likely mediating the consequences for the offspring. From weaning on, all offspring was fed a control diet; from 12 to 24 weeks of age, half of the offspring received a western-style diet (WSD). The combination of LPS-exposure and WSD resulted in hyperphagia and increased body weight and body fat mass in the female offspring. This was accompanied by changes in glucose tolerance, leptin and insulin levels and gene expression in liver and adipose tissue. In the hypothalamus, expression of genes involved in food intake regulation was slightly changed. We speculate that altered food intake behaviour is a result of dysregulation of hypothalamic signalling. Our results add to understanding of how maternal inflammation can mediate long-term health consequences for the offspring. This is relevant to many gestational complications with a pro-inflammatory reaction in place.

## Introduction

Circumstances during early life, even before conception, have a major impact on adult cardiovascular, metabolic and neurological health. This is described as fetal programming or the developmental origins of adult health and disease (DOHaD) hypothesis^1,2^. Here, we examine how prenatal exposure to maternal inflammation, combined with a western-style diet (WSD) later in life, influences the offspring’s metabolic, cardiovascular and neurological outcome.

Pregnancy is an important window to promote life-long health, however the intrauterine environment can be altered by many factors, including maternal nutrition, stress, and pregnancy complications^3,4^. An aberrant intrauterine environment can alter fetal growth, with both macrosomia or growth restriction as a result. Preeclampsia (PE), especially the early onset type, is often accompanied by intrauterine growth restriction, while, on the other hand, maternal obesity and gestational diabetes mellitus can lead to neonatal macrosomia. Surprisingly, life-long consequences for the offspring of these different complications are comparable, inducing similar adverse cardiovascular development and metabolic dysregulation in adulthood^5–8^. This suggests shared mechanisms being at play. Many gestational complications, including gestational diabetes mellitus, PE, and maternal obesity, come with a pro-inflammatory profile for the duration of the complication, which is likely an important mediator in fetal programming^9,10^. Next to these low-grade inflammatory changes, also acute inflammatory insults, such as bacterial infections, can take place during pregnancy. Acute inflammation can lead to preterm labour and stillbirth, but can also affect the developing organs and thereby long-term health of the offspring^11^.

Inflammation is accompanied by major changes in maternal circulating factors and placental biology, which will influence fetal programming. Cytokines are known to regulate normal placenta development, including trophoblast invasion, while aberrant cytokine signalling can lead to adverse placental development, perfusion and expression of nutrient transporters^9,12^. This can result in altered growth patterns with all its long-lasting consequences. Moreover, inflammation enhances the level of oxidative stress in both mother and fetus^13,14^. Reactive oxygen species are able to influence fetal programming by causing DNA damage and protein and lipid peroxidation^15^. Furthermore, cytokines and maternal immune cells can also be transported through the placenta to directly influence the fetus. However, this is highly cell-, cytokine-, and trimester-dependent and not fully understood yet, in mice nor in human^10^.

A well-established mouse model for maternal inflammation is the administration of lipopolysaccharides (LPS), the main component of the outer cell membrane of gram-negative bacteria. LPS administration induces a T helper 1-response, with an increase in pro-inflammatory cytokines and chemokines, including IL-6, TNFα and MCP-1. The response to LPS is most prominent in the first hours after injection and diminishes after 24–48 h^16^. In pregnant rodents, the pro-inflammatory response following LPS administration was observed in maternal and fetal plasma and in amniotic fluid^17,18^.

Several rodent studies showed that fetuses exposed to changes in maternal inflammatory factors show phenotypical aberrancies at birth and later in life. Low-dose LPS exposure at the end of gestation results in intrauterine growth restriction, followed by catch-up growth, in females^19^, while mid-gestational LPS in rats leads to myocardial fibrosis and elevated blood pressure and, in males, to metabolic dysregulation^20–22^. Dosing of LPS is critical, as high doses induce a severe maternal response leading to fetal death^23^. Research in our lab showed that combined prenatal exposure to low-dose LPS and soluble fms-like tyrosine kinase-1 (sFlt-1), an anti-angiogenic factor, in mice, leads to growth restriction in the fetus, with brain sparing taking place in the males only. This was accompanied by changes in the plasma metabolome in both dam and male and female offspring^24^. Thus, consequences of LPS during pregnancy are present at birth and later in life, and vary widely by dose, timing, and cofactors.

Maternal inflammation has been described as a risk factor for neurological diseases, and dysfunction of mostly microglia, but also other cell types including astrocytes, has been suggested to contribute to neurodevelopmental disorders as RETT syndrome, schizophrenia, and autism spectrum disorders^25,26^. Microglia, the innate immune cells of the central nervous system, are involved in the regulation of immune defence and central nervous system-homeostasis, by continuously monitoring their environment and orchestrating synaptic formation and modification^27,28^. From embryonic development onwards, microglia form a self-sustaining cell population without repopulation^28^. Therefore, during the embryonic period, neurodevelopment, and more specifically microglia, might be greatly affected by maternal immune activation with long-lasting consequences for the offspring^25,29^.

**Figure 1:**
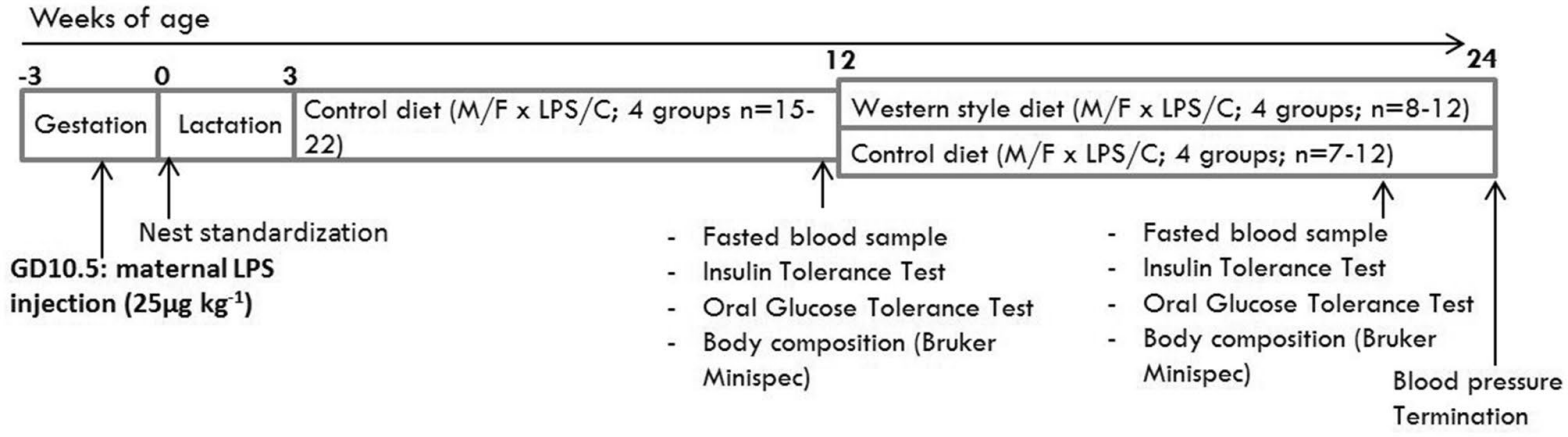
Experimental set-up. Pregnant C57Bl/6J mice were injected with 25 µg kg^−1^ LPS to induce inflammation at gestational day 10.5. An sFlt-1 adenovirus injection at GD 8.5 failed and is therefore not mentioned in the scheme. Half of the offspring received a western-style diet from 12 to 24 weeks of age. Offspring characteristics were analysed at 12 and 24 weeks of age.

Thus, maternal inflammation is likely to program long-term cardiovascular, neurologic, and metabolic health of the offspring in many ways. Because inflammation is an important factor in major gestational complications, it is important to elucidate the programming mechanisms involved in the fetus and the neonate. However, in epidemiological data, it is hard to distil the fetal consequences of inflammation from all other influencing factors during complicated pregnancy. Because adverse fetal organ development might also decrease metabolic capacity and alter coping with inflammation, WSD administration in the offspring is likely to exaggerate the consequences of gestational inflammation. Furthermore, considering the consumption of sugar- and fat rich diets in the human population, WSD administration to the offspring increases the clinical relevance of this model. In this study, the consequences of maternal inflammation, induced by a single, low-dose, LPS injection, in combination with a WSD later in life on cardiovascular, metabolic and neurological features of the offspring are examined (Fig. 1).

## Results

### sFlt-1 adenovirus injection did not increase plasma sFlt-1 levels

In this study, we followed an established protocol for generating well-characterized experimental preeclampsia^24^. This protocol consists of the injection of an adenovirus encoding for sFlt-1 at GD 8.5 and an LPS injection at GD 10.5, with controls receiving empty adenovirus and PBS, and PE is characterized by a twofold increase in sFlt-1 levels^24^. Unfortunately, in this study, sFlt-1 levels were not increased in our treatment group. Further testing of the sFlt-1 adenovirus in non-pregnant females revealed that its effectiveness had diminished since the previous use (Supplementary Fig. S1 online). Therefore, we conclude that the current sFlt-1 adenovirus preparation is inactive and consider this study to be modelling maternal inflammation only rather than preeclampsia.

### LPS-treatment leads to reduced pregnancy success and oxidative stress in LPS-exposed dams

Out of the 14 pregnant dams that received 25 µg kg^−1^ LPS at gestational day (GD) 10.5, six did not deliver a nest, which was preceded by a reduced weight gain at GD 15.5. Nest sizes and body weight of the LPS-treated dams that did deliver a nest were not significantly different from the control dams. It was noticeable that the three dams with a small litter showed repressed growth curves after the LPS injection, which indicates resorption of a part of their litter. Maternal weight gain at GD 15.5 per delivered pup showed no indication of fetal growth restriction (Supplementary Fig. S2 online).

**Figure 2:**
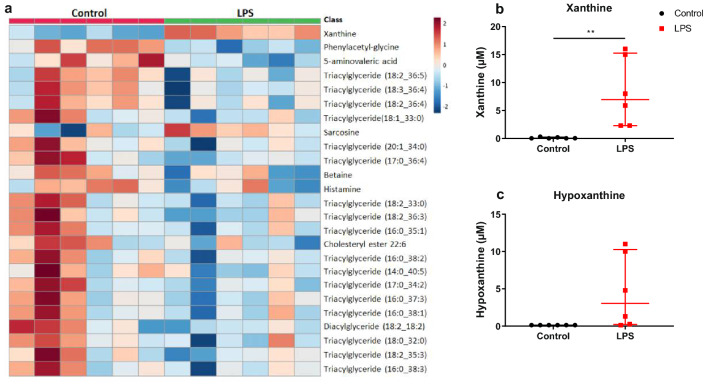
Plasma metabolomics at gestational day 15.5 of dams exposed to LPS at gestational day 10.5 and their controls. Heatmap of the top-25 changed metabolites based on t tests. Colour intensity in the red spectrum shows an increase of the metabolite, while colour intensity in the blue spectrum shows a decrease (**a**). Levels of xanthine (**b**) and hypoxanthine (**c**). Data in (**b**,**c**) are presented as median (interquartile range). **p < 0.01. *LPS* lipopolysaccharides.

To evaluate whether low dose LPS has metabolic consequences potentially influencing the fetuses, the maternal plasma metabolome was characterized at GD 15.5 (Fig. 2a). A significant increase of xanthine in LPS-treated dams was found (p < 0.01; Fig. 2b). Because of its relevance for xanthine metabolism, levels of hypoxanthine are also presented. However, this data was not statistically analysed because all samples in the control group, but none in the LPS group, were below the limit of detection (Fig. 2c).

### Increased body weight, fat mass and food intake in LPS + WSD female offspring

**Figure 3:**
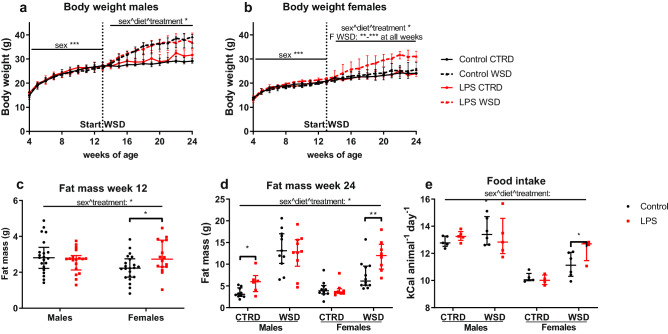
Offspring body weight, fat mass and food intake after in utero maternal inflammation exposure combined with a WSD later in life. Body weight from 4 to 24 weeks of age in males (**a**) and females (**b**) and fat mass at week 12 (**c**) and 24 (**d**). Average daily food intake of the offspring, measured in week 22–24, multiplied by the caloric content of the diet and divided by the number of days and animals per cage (**e**). Analysed using 3-way (repeated measures) ANOVA, data are presented as median (interquartile range). *p < 0.05; **p < 0.01; ***p < 0.001. *CTRD* control diet, *WSD* western-style diet, *LPS* lipopolysaccharides.

Offspring were fed a control diet (CTRD) until 12 weeks of age (WK12). In this time frame, treatment did not affect body weight (Fig. 3a,b). However, at WK12, LPS-exposed females had a significantly higher body fat mass (p < 0.05; Fig. 3c). When exposed to the WSD, LPS-exposed females gained more weight than their controls (p < 0.01 or p < 0.001 at all weeks), which was also reflected in an elevated body fat mass at WK24 (p < 0.01; Fig. 3b,d). LPS CTRD males also showed a significantly increased fat mass (p < 0.05), but not body weight, at WK24 (Fig. 3a,d). When corrected for body weight, all observed fat mass differences remained. Average daily food intake at WK22-24 was significantly higher in LPS WSD females compared to their WSD controls (p < 0.05; Fig. 3e).

To analyse growth patterns, several metabolic and cardiovascular relevant tissues were weighed at termination. After correcting for body weight, liver, heart, and kidney weight were only significantly changed by diet; liver was relatively heavier in WSD mice, while kidney and heart were relatively lighter. After correcting for body weight, gonadal and inguinal white adipose tissue weight remained significantly heavier in the LPS-treated WSD females compared to their controls (p < 0.05 and p < 0.01; Supplementary Table S1 online). No significant LPS-induced effect on systolic and diastolic blood pressure was found (Supplementary Fig. S3 online).

### Sex-, diet- and treatment-specific metabolic differences

**Figure 4:**
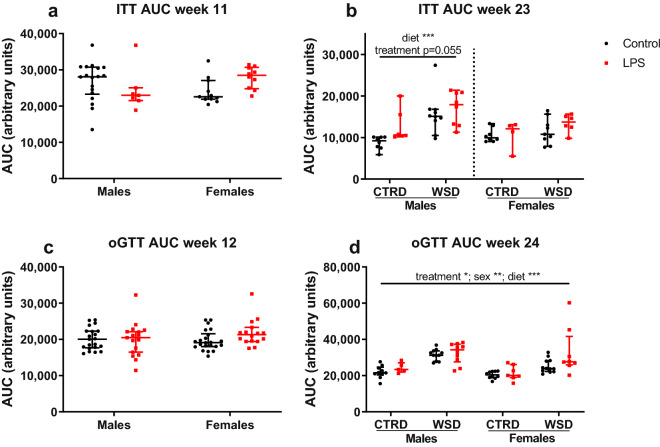
Area under the curve of blood glucose levels during insulin tolerance and oral glucose tolerance tests in offspring exposed to maternal inflammation in utero combined with a WSD later in life. Insulin tolerance was examined in week 11 (**a**) and 23 (**b**) and glucose tolerance was examined in week 12 (**c**) and 24 (**d**). Area under the curve was calculated using the trapezoidal method. Analysed using 2-way (week 11/12) and 3-way (week 23/24) ANOVA, data are presented as median (interquartile range). *p < 0.05; **p < 0.01; ***p < 0.001. *CTRD* control diet; *WSD* western-style diet, *LPS* lipopolysaccharides.

Metabolic consequences of LPS-exposure were tested with insulin- and glucose tolerance tests. Insulin tolerance testing at WK11 showed no sex- or treatment-based differences at the time points measured or in area under the curve (Fig. 4a). At WK23, LPS treatment tended to worsen insulin tolerance, as measured by the area under the curve, in males (p = 0.055; Fig. 4b). Hypoglycaemic mice that needed glucose administration are excluded from analysis, which is justified as their number did not differ per group. No differences were found in glucose tolerance at WK12 (Fig. 4c). At WK24, male sex (p < 0.01), WSD (p < 0.001) and LPS treatment (p < 0.05) increased the area under the curve of the glucose tolerance test (Fig. 4d). Time point curves of insulin- and glucose tolerance tests are shown in Supplementary Figs. S4 and S5 online.

**Figure 5:**
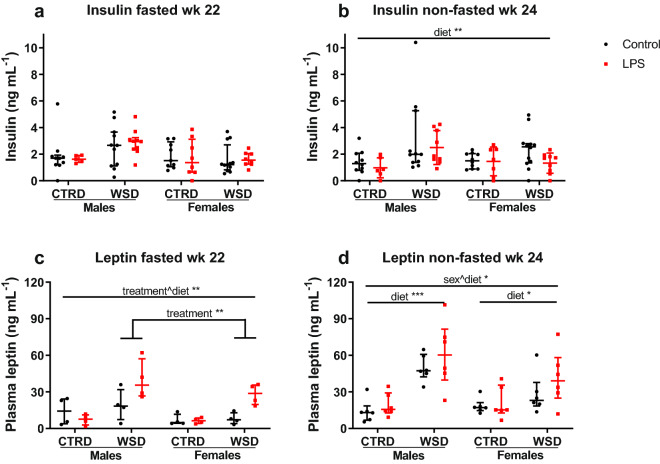
Fasted and non-fasted plasma insulin and leptin levels in mice exposed to maternal inflammation in utero combined with a WSD later in life. Fasting levels of plasma insulin (**a**) and leptin (**c**) in week 22 and non-fasting levels of insulin (**b**) and leptin (**d**) in week 24. Analysed using 3-way ANOVA, data are presented as median (interquartile range). *p < 0.05; **p < 0.01. *CTRD* control diet, *WSD* western-style diet, *LPS* lipopolysaccharides.

Fasting insulin in WK22 was unchanged, while non-fasted insulin was significantly increased by WSD (p < 0.01; Fig. 5a,b). In the fasted samples of WK22, leptin levels were significantly increased by LPS exposure in the WSD group only (p < 0.01), while in the non-fasted samples, leptin levels were increased by diet in both males (p < 0.001) and females (p < 0.05; Fig. 5c,d). Fasting levels of triglycerides were increased by LPS-treatment at WK10 (p < 0.01; Supplementary Fig. S6 online).

### LPS treatment changes gene expression in liver and white adipose tissue

**Figure 6:**
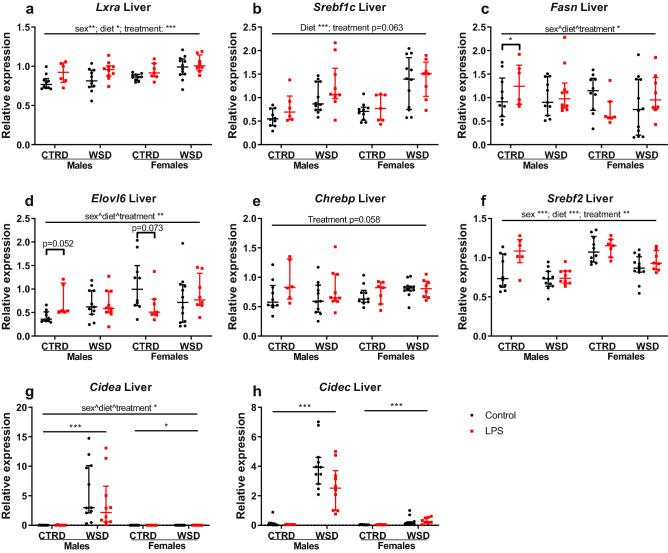
Hepatic gene expression in mice exposed to maternal inflammation in utero combined with a WSD later in life. Gene expression of *Lxra* (**a**), *Srebf1c* (**b**), *Fasn* (**c**), *Elovl6* (**d**), *Chrebp* (**e**), *Srebf2* (**f**), *Cidea* (**g)** and *Cidec* (**h**) are shown in this figure, expression of other genes is shown in Supplementary Table S2 online. Relative expressions are calculated using a standard curve and corrected for the relative expression of *36b4*. Analysed using 3-way ANOVA, data are presented as median (interquartile range). *p < 0.05; **p < 0.01; ***p < 0.001. *CTRD* control diet, *WSD* western-style diet, *LPS* lipopolysaccharides.

**Figure 7:**
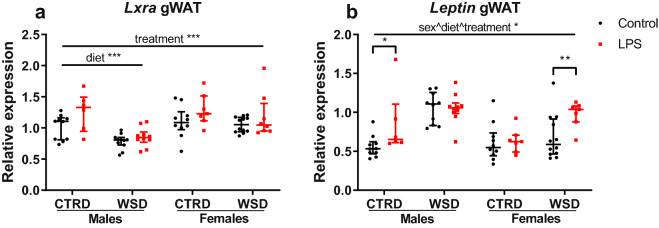
Relative gene expression in gonadal white adipose tissue of mice exposed to maternal inflammation in utero combined with a WSD later in life. Gene expression of *Lxra* (**a**) and *Leptin* (**b**) are shown in this figure, expression of other genes is shown in Supplementary Table S3 online. Relative expressions are calculated using a standard curve and corrected for the relative expression of β-actin. Analysed using 3-way ANOVA, data are presented as median (interquartile range). *p < 0.05; **p < 0.01; ***p < 0.001. *CTRD* control diet, *WSD* western-style diet, *LPS* lipopolysaccharides.

Fatty acid metabolism was analysed in liver and gonadal white adipose tissue (gWAT) using gene expression of a number of key genes involved in fatty acid metabolism^30,31^. In liver, increased gene expression by LPS-treatment was found in *Lxra* (p < 0.001), *Srebf2* (p < 0.01), *Chrebp* (p = 0.058) and *Srebf1c* (p = 0.063) expression. Sub-analysis of three-way interactions showed a significant difference between LPS and controls in CTRD males for *Fasn* and a trend in CTRD males and females for *Elovl6*. *Cidea* and *Cidec*, involved in hepatic steatosis^32,33^, showed a dramatic increase in WSD males (p < 0.001 for both genes compared to CTRD males) and a more modest increase in WSD females (p < 0.05 and p < 0.001, respectively, compared to CTRD females), irrespective of LPS treatment (Fig. 6). WSD-induced increased gene expression was found for *Ppara*, *Cpt1a*, *Scd1* and *Pparg*, no treatment- diet- or sex-induced changes were found for *Acaca* (Supplementary Table S2 online). In gWAT, *Lxra* expression was significantly higher (p < 0.001) in the LPS-treated animals compared to the controls. Sub-analysis of a three-way sex^diet^treatment interaction showed a significant difference between LPS and controls in CTRD males and WSD females for *Leptin* (Fig. 7). Diet- and/or sex-induced differences were found for *Fasn, Srebf2, Pparg, Zfp423, Scd1, Chrebf, Cdkn2a, 11bhsd1* and *Nr3c1* (Supplementary Table S3 online).

### No DNA methylation alterations of hepatic *Fasn*, *Lxra* and *Srebf2*

Effects of LPS treatment on DNA methylation was analysed in the promoter regions of *Fasn, Lxra* and *Srebf2* in DNA isolated from the liver. No treatment-induced differences were found in average methylation (Supplementary Fig. S7 online) nor in methylation of the individual CpG positions for all three genes. *Fasn* DNA methylation and gene expression were correlated (pearson correlation − 0.287, p = 0.019), while for *Lxra* and *Srebf2* no such correlation was found.

### Several hypothalamic genes are affected by sex, diet or LPS treatment

**Figure 8:**
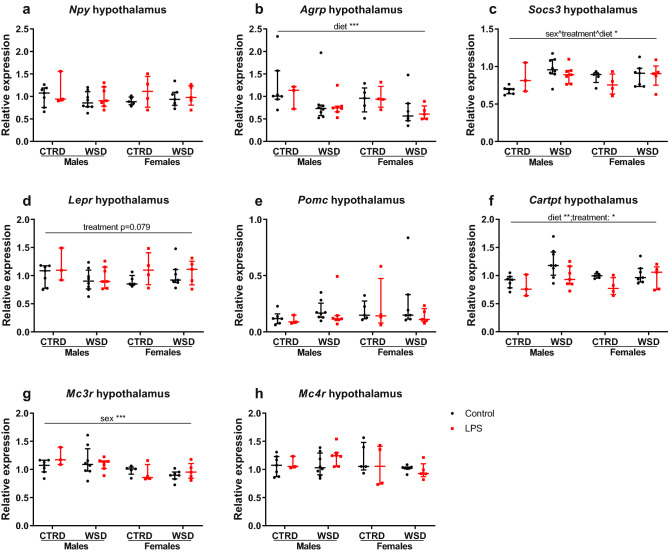
Hypothalamic expression of (an)orexigenic genes in mice exposed to maternal inflammation in utero combined with a WSD later in life. Relative expression of orexigenic genes *Npy* (**a**) and *Agrp* (**b**), and of the anorexigenic genes *Socs3* (**c**), *Lepr* (**d**), *Pomc* (**e**), *Cartpt* (**f**), *Mc3r* (**g**), and *Mc4r* (**h**). Relative expressions are calculated using a standard curve and corrected for the average relative expression of *Hprt* and *β-actin*. Analysed using 3-way ANOVA, data are presented as median (interquartile range). *p < 0.05; **p < 0.01; ***p < 0.001. *CTRD* control diet, *WSD* western-style diet, *LPS* lipopolysaccharides.

**Figure 9:**
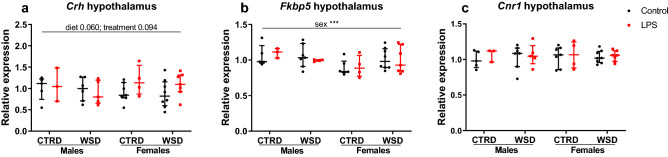
Hypothalamic gene expression in mice exposed to maternal inflammation in utero combined with a WSD later in life. Relative gene expression of *Crh* (**a**), *Fkbp5* (**b**), and *Cnr1* (**c**). Relative expressions are calculated using a standard curve and corrected for the average relative expression of *Hprt* and *β-actin*. Analysed using 3-way ANOVA, data are presented as median (interquartile range). ***p < 0.001. *CTRD* control diet, *WSD* western-style diet, *LPS* lipopolysaccharides.

Because leptin signalling to the hypothalamus is a major regulator of food intake, gene expression and DNA methylation of the key (an)orexigenic genes in the hypothalamus were analysed^34^. Some sex-, diet- and treatment-based changes were found in key orexigenic (*Npy* and *Agrp*) and anorexigenic (*Socs3, Lepr, Pomc, Cartpt, Mc3r* and *Mc4r*) genes in the hypothalamus (Fig. 8). LPS-treatment significantly lowered *Cartpt* expression (p < 0.05), and a trend for increased *Lepr* expression was found (p = 0.079), which seems to be most pronounced in the females. Hyperphagia and excessive weight gain were only found in LPS/WSD females, not in LPS/CTRD females, indicating that, on top of LPS-exposure, diet palatability or hedonic eating could play a role. This led us to hypothesize that these LPS females show comfort-seeking behaviour, resulting in increased intake of a palatable diet^35^. Therefore, next to the (an)orexigenic genes, gene expression of *Crh, Cnr1* and *Fkbp5,* genes relevant to food intake behaviour^36–38^, was measured. For the hypothalamic genes involved in food intake behaviour, a trend for increased *Crh* by LPS-treatment was found (p = 0.094; Fig. 9), again most pronounced in the female offspring.

DNA methylation analysis of a number of CpG positions in the promoter region of *Crh*, *Pomc* and *Lepr* and in the *Lepr* exon showed no LPS-associated effects for the average of all CpG positions per gene. Changes in individual CpG positions were not consistent, as methylation of one CpG position in the *Lepr* promoter was increased in LPS-exposed males, but decreased in females, while methylation of a neighbouring CpG position was increased in LPS-exposed males. A trend for LPS-associated increased DNA methylation of a single *Pomc* CpG position was found (Supplementary Fig. S8 online).

### LPS treatment changes cortical gene expression, depending on sex and diet

Cortical expression of microglia and astrocyte activation and homeostasis-related genes, showed treatment and sex-induced differences (Table 1). No changes were found in general activation markers *Apoe* and *Cxcl10*. Marker for microglia homeostasis *Vista* was generally decreased by LPS-treatment (p < 0.01) and microglia homeostasis markers *P2ry12* and *Tmem119* were decreased by LPS-treatment in the male CTRD (p < 0.05 and p < 0.001) and female WSD (p < 0.05 for both genes) group only. Controversially, microglia activation-markers *Tyrobp* and *Ctsd* were also found decreased by LPS in the male CTRD group (p < 0.05 for both). Astrocyte homeostasis marker *Aqp4* was decreased in the male CTRD and female WSD group (p < 0.05 for both), as well as astrocyte activation markers *Gfap* (M CTRD p < 0.01; F WSD p = 0.054) and *Axl* (M CTRD p < 0.001; F WSD p = 0.067). Astrocyte homeostasis marker *Slc1a2* showed LPS-induced increase in the female CTRD group (p < 0.05) and astrocyte activation marker *Bdnf* was increased in the male WSD group (p < 0.05).

**Table 1:** Expression in the cortex of astrocyte- and microglia related genes in mice exposed to maternal inflammation in utero combined with a WSD later in life.

	M CTRD Control	M CTRD LPS	M WSD Control	M WSD LPS	F CTRD Control	F CTRD LPS	F WSD Control	F WSD LPS	Significance
**General activation**									
*Cxcl10*	0.96 (0.74–1.28)	0.96 (0.42–1.46)	0.99 (0.68–1.12)	0.70 (0.55–1.23)	0.70 (0.40–0.92)	0.58 (0.42–1.10)	1.05 (0.51–1.43)	0.94 (0.45–1.51)	
*Apoe*	1.21 (1.12–1.31)	1.13 (0.74–1.21)	1.21 (1.05–1.34)	1.10 (0.96–1.15)	1.10 (1.02–1.20)	1.30 (1.00–1.40)	1.26 (1.09–1.35)	1.06 (0.96–1.54)	
**Microglia homeostasis**									
*P2ry12*	1.24 (1.11–1.47)	0.73 (0.52–1.21)	1.14 (1.09–1.32)	1.27 (1.11–1.36)	1.06 (0.95–1.29)	1.41 (1.08–1.61)	1.24 (1.17–1.57)	1.08 (0.82–1.22)	M CTRD*; F WSD*
*Cx3cr1*	0.98 (0.84–1.10)	0.84 (0.74–1.04)	0.94 (0.89–1.07)	0.91 (0.85–1.07)	1.01 (0.87–1.13)	1.01 (0.92–1.15)	0.96 (0.90–1.22)	0.90 (0.86–1.00)	
*Tmem119*	1.06 (0.97–1.21)	0.56 (0.40–0.82)	1.03 (0.96–1.22)	0.95 (0.90–1.10)	0.96 (0.69–1.10)	1.40 (0.78–1.45)	1.27 (1.22–1.31)	0.92 (0.60–1.28)	M CTRD***; F WSD*
*Vista*	0.99 (0.83–1.00)	0.68 (0.45–0.76)	0.93 (0.73–1.06)	0.87 (0.83–1.06)	0.95 (0.86–1.03)	1.08 (0.85–1.18)	1.08 (1.02–1.19)	0.82 (0.70–0.93)	Sex**; treatment*
**Microglia activation**									
*Cd68*	1.20 (1.06–1.31)	1.05 (0.70–1.35)	1.17 (1.13–1.37)	1.06 (0.98–1.37)	1.13 (0.93–1.22)	1.29 (1.07–1.51)	1.30 (1.18–1.43)	1.25 (1.03–1.38)	
*Tyrobp*	1.09 (0.92–1.17)	0.74 (0.41–1.02)	1.03 (0.91–1.21)	1.05 (0.97–1.25)	1.01 (0.76–1.20)	1.12 (0.98–1.51)	1.09 (0.92–1.38)	0.88 (0.83–1.15)	M CRTD*
*Ctsd*	1.19 (1.02–1.31)	0.93 (0.47–1.02)	1.13 (0.95–1.21)	1.12 (0.99–1.28)	1.09 (0.89–1.25)	1.24 (1.06–1.36)	1.30 (1.23–1.33)	1.09 (0.96–1.35)	M CTRD*
*H2aa*	0.69 (0.49–0.88)	2.77 (1.62–11.56)	0.89 (0.47–2.04)	0.51 (0.35–0.83)	0.51 (0.25–0.84)	0.83 (0.57–3.26)	0.80 (0.67–1.25)	0.99 (0.83–1.15)	M CTRD***
**Astrocyte homeostasis**									
*Fgfr3*	1.06 (0.95–1.18)	1.01 (0.67–1.080	1.06 (0.87–1.20)	1.21 (0.99–1.25)	1.01 (0.89–1.26)	1.33 (0.98–1.41)	1.18 (1.03–1.28)	1.03 (0.85–1.17)	
*Aqp4*	1.20 (1.02–1.37)	0.80 (0.60–1.06)	1.05 (0.94–1.28)	1.12 (0.83–1.24)	1.17 (1.05–1.22)	1.24 (1.14–1.64)	1.23 (1.16–1.42)	0.97 (0.84–1.20)	M CTRD*; F WSD*
*Slc1a2*	1.08 (1.03–1.16)	0.90 (0.55–1.15)	1.08 (0.97–1.18)	1.09 (0.99–1.28)	1.01 (0.86–1.14)	1.25 (1.10–1.35)	1.16 (1.07–1.24)	0.97 (0.80–1.17)	F CTRD*
**Astrocyte activation**									
*Bdnf*	1.10 (0.99–1.27)	0.99 (0.96–1.28)	1.04 (0.92–1.25)	1.36 (1.19–1.42)	1.11 (0.92–1.38)	1.27 (1.02–1.33)	1.17 (1.06–1.29)	1.09 (0.90–1.22)	M WSD*
*Axl*	1.15 (1.06–1.32)	0.49 (0.31–0.84)	1.17 (0.82–1.30)	1.05 (0.90–1.16)	1.03 (0.88–1.14)	1.19 (0.81–1.25)	1.13 (1.03–1.28)	0.84 (0.79–1.15)	M CTRD***; F WSD 0.067
*Gfap*	1.21 (0.89–1.21)	0.64 (0.42–1.07)	0.92 (0.65–1.17)	1.12 (0.86–1.30)	0.95 (0.72–1.18)	1.20 (0.76–1.33)	1.25 (1.15–1.34)	1.00 (0.73–1.09)	M CTRD**; F WSD 0.054

Relative gene expressions are calculated using a standard curve and corrected for the average relative expression of *Gapdh*. Analysed using 3-way ANOVA, data are presented as median (interquartile range). *p < 0.05; **p < 0.01; ***p < 0.001.

*F* female, *M* male, *CTRD* control diet, *WSD* western-style diet, *LPS* lipopolysaccharides.

## Discussion

Evidence from human and animal studies indicates that maternal inflammation during gestation is associated with many adverse long-term health outcomes for the offspring^10^. Our study shows that already a single hit with a low dose of LPS at mid gestation has long-lasting consequences for offspring’s body weight, food intake, and associated gene expression, most pronounced in females on a western-style diet. The increased fat mass and body weight are associated with metabolic differences in LPS WSD females compared to the control WSD females, including impaired glucose clearance and increased fasting leptin. Effects in WSD and CTRD males and CTRD females were more subtle or absent.

Disturbed glucose- and fat metabolism can be a consequence of altered gene expression in liver or white adipose tissue. With colleagues, we have found that prenatal interventions can alter gene expression and DNA methylation of *Lxra*, resulting in dysregulated lipid and cholesterol metabolism^39,40^. In accordance with these studies, in our study LPS-exposure increased the expression of *Lxra* in both liver and adipose tissue, and the expression of *Srebf2* in liver only, with the effects being most pronounced in CTRD males. Furthermore, *Leptin* expression in white adipose tissue was increased in LPS-exposed CTRD males and WSD females, the same groups that had significantly more fat mass than their controls. *Lxra, Fasn* and *Srebf2* DNA methylation and expression of other hepatic lipogenic or lipolytic genes were unchanged by LPS, indicating that LPS-induced in utero programming of hepatic lipid and cholesterol metabolism is unlikely. Even though the LPS WSD females gained more weight and body fat than their controls, hepatic expression of *Cidea* and *Cidec*, markers of large lipid droplet formation and hepatic steatosis^32,33^, were not affected by LPS treatment. Off note, gene expression of those markers was dramatically increased by WSD in males, and more modestly in females.

Increased fat mass and body weight occurs when energy intake exceeds energy expenditure. Both increased intake and decreased expenditure have been found sensitive for fetal programming by adverse in utero conditions^41,42^. LPS-treatment did increase food intake and body weight in the WSD females only. As increased food intake is only observed in a sex- and diet-specific group, a general LPS-caused decrease in energy expenditure is probably not causative, as a general LPS-induced elevation in food intake would then be expected. Taken together, it is likely that the observed metabolic differences are caused by increased body fat mass, and the increased fat storage in the LPS WSD females is the result of increased food intake. Therefore, we investigated possible mechanisms for increased food intake.

Leptin sensitivity and energy balance-related hypothalamic pathways are known to be vulnerable for fetal programming^34^. This can be by epigenetic alterations, including DNA methylation, histone modifications and microRNAs, but also structurally, by altered neuronal projections^43^. Male and female mice and rats exposed to maternal obesity throughout gestation and lactation show increased food intake and body weight, with impaired leptin signalling caused by high neonatal leptin levels being a likely mechanism^44,45^. Hypoxia during late gestation results in hyperphagia in male rat offspring, which is exaggerated by a high fat diet. The proposed mechanism in the hypoxic rat model is compromised development of hypothalamic arcuate nucleus projections^46^. In the present study, LPS-associated differences in fasting leptin levels and gene expression of associated genes in the whole hypothalamus were found, indicating that leptin signalling is at least partially mediating the observed phenotype. Because hypothalami were collected in the fed state, when leptin levels are similar between the treatment groups, leptin action on the hypothalamus could be compared between LPS and control groups. In the fed state, expression of the leptin receptor tended to be increased, most pronounced in the WSD females, while the expression of *Cartpt*, an anorexigenic peptide which is induced by leptin, was decreased by LPS. In ovaries, *Cartpt* is known to be vulnerable for fetal programming by gestational diabetes mellitus^47^. It might be speculated that a decreased anorexigenic *Cartpt* signal is involved in hyperphagia.

Besides programming of energy balance, food preference and reward-related eating is also subject to fetal programming in the hypothalamus^35^. LPS administration induces secretion of stress-related factors, such as corticosteroids, to which fetuses are exposed^48^. Early-life stress is known to lead to increased food intake and body weight gain, as well as adverse metabolic parameters, later in life. Dysregulation of hypothalamic–pituitary–adrenal (HPA) axis, where stress and food intake are integrated, is a proposed mechanism^49,50^. Analysis of the whole hypothalamus showed a trend for LPS-induced increased gene expression of *Crh*, a key gene in the HPA axis. This is especially observed in the WSD females, which is the group that showed the most pronounced dysregulation of food intake and body weight. Taken together, hypothalamic gene expression data indicates that the hypothalamus is involved in LPS-induced hyperphagia. Differences might however be blunted because of the low number of samples for this specific tissue and because of the moment of collection of the hypothalami, which was in the first half of the light phase. Considering that food intake is generally suppressed in the light phase and the major part of food intake takes place in the dark phase, differences in food intake and its hypothalamic regulation are expected to be more pronounced in the dark phase. For analysis of a delicate process as food intake regulation, one would prefer to have multiple time points and possibly also dietary challenges or a leptin resistance test. Data from this study indicates a dysregulation of hypothalamic signalling, however no conclusions on the mechanisms can be drawn.

The observed fetal programming can be a direct consequence of short-term exposure to the LPS-induced pro-inflammatory cytokines and cells, but also other mediators are possible. Analysis of the plasma metabolome at GD 15 showed that LPS has long-term consequences on oxidative stress after the peak inflammatory response at GD 10. We found a drastic increase in xanthine levels, and associated hypoxanthine levels were also increased. Xanthine and hypoxanthine are degradation products of purine, which are induced by hypoxic tissues and lead to the production of reactive oxygen species, and LPS is known to activate this pathway^51,52^. There were no obvious metabolomics differences between dams with and without fetal resorptions, suggesting that the resorptions do not influence maternal status. The metabolomics data show that the LPS-induced phenotype in the offspring can be a direct consequence of either the peak inflammation or the longer-term presence of oxidative stress, or a combination of both.

Interestingly, hyperphagia and increased body weight and fat mass are most pronounced in the LPS WSD female offspring, while LPS CTRD males show minor, non-significant increases. The moment of action of mechanisms behind this sex-specific difference can be either prenatal or postnatal. Prenatally, insults can sex-specifically influence placental development and transport, resulting in differential fetal exposure^53^. This is for instance the case for glucocorticoids, to which female fetuses are more sensitive, partly because testosterone inhibits glucocorticoid-regulated pathways^54^. Postnatally, an interaction between programmed factors and intrinsic differences between males and females, such as sex hormones or eating behaviour, can lead to sex-specific phenotypes. In this case, the male-like eating behaviour in LPS-exposed females could originate in changes in the hypothalamic–pituitary–gonadal axis, of which oestrogen is a key player^55^. An alternative suggestion is that LPS exposure preferably increases stress-related, hedonic eating, but in males WSD exposure rapidly leads to body weight gain, irrespective of prenatal treatment, which might have overruled the possible LPS-induced effects on food intake in the WSD males. This female-specific programming is remarkable, as in many models of fetal programming the males are more affected than the females^20,33^.

With respect to the microglia and astrocytes, we hypothesized an activation by WSD, with alterations by in utero preconditioning with LPS. However, we did not detect any significant changes in the expression of pro- and anti-inflammatory response genes of both microglia and astrocytes in the cortex after 3 months of WSD. We did find an LPS-associated decrease in astrocyte and microglia homeostasis and activation marker genes in CTRD males and WSD females, which is incoherent as one would expect those classes of genes to act in opposite directions. Both beneficial and unfavourable effects of LPS preconditioning were reported previously. More specifically, LPS-preconditioned microglia have been reported to have both neuroprotective properties^56,57^, as well as negative effects on learning and memory^58,59^. Besides, Schaafsma et al. showed that embryonic exposure to LPS results in a significantly attenuated microglial response to a second LPS challenge in adulthood^25^. The lack of coherent differences in this study might be due to the duration of the WSD, the dose of the LPS, the length of LPS preconditioning, or the period between LPS preconditioning and the WSD. Furthermore, we analysed whole cortex rather than isolated astrocytes and microglia.

The strengths of our study are that we provide long-term and well-controlled data on the impact of gestational LPS on offspring’s health, while the interaction with sex and WSD as a second hit are examined. Furthermore, a broad selection of tissues and pathways are examined. Weaknesses are the lack of neonatal and behavioural data, and the analysis of whole hypothalamus and cortex instead of separate nuclei. Furthermore, in order to disturb the pregnant dams as little as possible, no sequential blood samples were taken after LPS injection, so the exact duration of the LPS-induced inflammation cannot be reported.

In conclusion, here we report increased food intake, body weight and body fat mass in female mice exposed to maternal inflammation in utero and a western–style diet later in life. This is relevant to many gestational complications, as inflammation likely plays an important role in fetal programming. Analyses of possible pathways showed LPS-induced gene expression differences in white adipose tissue, liver, and hypothalamus, with changes in liver and white adipose tissue likely being a consequence of increased body weight, while changes in the hypothalamus could be causative for the observed hyperphagic phenotype. Our results add to understanding of how maternal inflammation can mediate long-term health consequences for the offspring.

## Materials and methods

### Animals and housing—dams

For the F0, male and female C57Bl/6J mice (Charles River, France) were housed individually with a 12 h light/dark cycle, lights on at 7 am, and water and chow (RMH-B, AB Diets) available ad libitum*.* From 12 weeks of age onwards, female oestrus cycle was monitored by vaginal cytology^60^. At pro-oestrus, 1 or 2 females were housed together with one male for overnight mating, and pregnancy was confirmed by presence of a vaginal plug the next morning, which was then considered GD 0.5. At GD8.5, pregnant females were randomly assigned to the treatment or control group, with the treatment group receiving 1 × 10^9^ sFlt-1 adeno virus units in 100 µL PBS, injected retro-orbitally. The control group underwent the same treatment, using empty adenovirus. This adenovirus injection turned out to be ineffective, as shown in the results section. At GD 10.5, the treatment group (n = 14) received an intraperitoneal injection of 25 µg kg^−1^ LPS (E. coli 0111:B4, Sigma-Aldrich) in sterile saline (100 µL). The control group (n = 11) received sterile saline. A blood sample was taken from the tail at GD 15.5, and sFlt-1 concentrations were measured using ELISA (R&D Systems Mouse VEGFR1/Flt-1 Quantikine ELISA Kit, #MVR100). All dams delivered at GD 19. Animal experiments were approved by the Dutch national ethical board and the institutional ethical committee of the University of Groningen, and are in accordance with the European Convention for the Protection of Vertebrate Animals used for Experimental and other Scientific Purposes (Council of Europe No 123, Strasbourg 1985).

### Animals and housing—offspring

At postnatal day one to three, nests were standardised to 5 or 6 pups per dam, containing at least 1 male from a different dam, to normalize rearing and accommodate later life pair-housing. Besides standardisation, dams and litters were left untouched until weaning at postnatal day 21. When available, 2 males and 2 females per dam were used for long-term experiments, which were later split over two different diet groups. The F1 was pair-housed, with each pair containing males or females from 2 different dams, unless a sufficient cage-mate from another litter was not available. Excessively fighting males were housed separately. Housing conditions were identical to the dams. From weaning until WK12, all animals were fed a semi-synthetic control diet (D13100302, Research Diets), ad libitum. At WK12, the groups were divided into a western-style diet (D12079B Research Diets) and a control diet subgroup, food again provided ad libitum (dietary composition shown in Supplementary Table S4 online). Males and females were analysed separately, which resulted in 8 experimental groups, as shown in the time line (Fig. 1).

### In vivo measurements

Body weight was recorded weekly. Food intake was measured at WK22, 23 and 24, and the caloric intake per animal per day was calculated. At WK10 and 22, a blood sample was taken after a 6-h morning fast (9 am–3 pm).

At WK11 and 23, all offspring underwent an intraperitoneal insulin tolerance test after a 4-h morning fast (9 am–1 pm). Insulin (NovoRapid insuline aspart, Novo Nordisk) was diluted in sterile PBS to 0.1 U mL^−1^ and injected in the peritoneum dosed at 0.5 U kg^−1^ for females and 12-week old males and at 0.75 U kg^−1^ for the 23-weeks old males. Blood glucose was measured via tail snipping before insulin injection and 15, 30, 45, 60, 90 and 120 min after insulin injection, using OneTouch Select Plus glucometer and strips (LifeScan). Animals showing signs of severe hypoglycaemia were rescued with a glucose injection. At WK12 and 24, all offspring underwent an oral glucose tolerance test after 10 h of overnight fasting (11 pm–9 am). A 20% glucose (Sigma #49139) solution in PBS was administered via oral gavage, dosed at 1 g kg^−1^ in 12-week old mice and 2 g kg^−1^ in 24-week old mice. Blood glucose was measured before glucose administration and at 15, 30, 60, 90 and 120 min after the treatment. Area under the curve was calculated using the trapezoidal method. At WK12 and 24, body composition was measured using a Bruker minispec LF90II Body Composition Analyzer. The minispec procedure consisted of 1 min of restrainment in a tube per measurement, without any anaesthesia.

Termination took place at WK24. Animals were anesthetized by isoflurane inhalation. Blood pressure in the abdominal aorta was measured using a fluid filled catheter attached to a Cardiocap/5 blood pressure monitor (Datex-Ohmeda), blood was taken by cardiac puncture. Heart, left kidney, liver, gonadal and inguinal white adipose tissue pads were weighed and collected by snap-freezing. Hypothalamus and whole cortex were collected by snap freezing.

### Plasma analyses

All blood samples were collected in EDTA-coated tubes and centrifuged at 2000*g* for plasma separation. sFlt-1 concentrations were assessed in maternal plasma, using a mouse sFlt-1 ELISA kit (R&D systems, MVR100). In the offspring, leptin (Crystal Chem #90030), and triglycerides (Roche Diagnostics, 11877771216) were measured in the fasted plasma samples of both time points and in the unfasted terminal sample. Samples for leptin analysis were selected at random. Insulin (Crystal Chem #90010 with mouse standard #90020) was measured in fasted plasma samples of week 22 and in unfasted terminal samples.

### Metabolomics

Plasma was obtained and stored as described above. In 10 µL samples of 12 dams, plasma metabolome was analysed by Biocrates Life Sciences AG, using their MxP Quant500 kit, at their facility. Levels of 630 metabolites from 26 compound classes, including a range of amino acids, bile acids, acylcarnitines, phosphaditylcholines and triglycerides, were quantified, by using a combination of flow injection analysis and liquid chromatography-based triple quadrupole mass spectrometry. Internal standards and a quality controls at three concentration levels were used to ensure reliable quantification.

### Molecular analyses

DNA and RNA isolation from liver, gonadal white adipose tissue and cortex took place using TRIzol reagent (Thermo Fisher Scientific) with Back Extraction Buffer, following manufacturer’s protocol. Cortex was homogenized before isolation to avoid region-specific effects. DNA and RNA isolation from whole hypothalamus took place using the AllPrep DNA/RNA Mini kit (Qiagen). DNA and RNA quantity was measured using Nanodrop. 1 µg of RNA was converted into cDNA and used for gene expression analysis by qPCR. In cortex and hypothalamus, gene expression was assessed using primers and SYBR Green Mastermix (Fisher Scientific). In liver and gWAT, primer–probe combinations and Taqman Fast Advanced Master Mix (Fisher Scientific) were used, except for *Cidea* and *Cidec* in liver, which were analysed using SYBR Green. All qPCR runs took place using the Quantstudio 3 hardware and accompanying software, following the protocol of the mastermix manufacturer. Relative expressions were calculated using standard curves and corrected by the relative expression of housekeeping genes. Housekeeping genes were selected per organ based on stable expression across the groups. Selected housekeeping genes are *Hprt* and *Actb* for hypothalamus, *Gapdh* for cortex, *36b4* for liver and *Actb* for gWAT. Housekeeping genes were analysed using the same mastermix as the gene of interest The full list of genes and primer sequences is shown in Supplementary Table S5 online.

For DNA methylation analysis, 500 ng of hepatic genomic DNA was treated with EZ DNA Methylation Gold kit (Zymo Research) to convert unmethylated cytosines to uracils. The regions of interest were amplified using PCR and DNA methylation was assessed using Pyromark Q48 (Qiagen), following manufacturer’s instructions. Sequences of forward and reverse primers, sequencing primers and sequences to analyse are shown in Supplementary Table S6 online.

### Statistics

Data was analysed using three-way Analysis of Variance (ANOVA), with sex, diet and treatment as the variables. When a significant interaction involving treatment was found, treatment-based differences between simple main effects were analysed at the level of the interaction. For 3-way interactions, pairwise comparisons for LPS-treated versus controls in all sex-diet subgroups took place. For 2-way interactions, pairwise comparisons between LPS-treated versus controls in sex- or diet-groups took place. In case of a sex-diet interaction, the simple main effects for diet were analysed for males and females separately. For data involving repeated measures (glucose- and insulin tolerance tests and body weight), a mixed 3-way ANOVA was employed to enable analysis of interactions with time. Differences between time points and time^diet and time^sex interactions are not expressed and both interactions are not further investigated. Interactions involving time^treatment were further analysed. All tests employed Sidak’s adjustment for multiple comparisons and, at the mixed ANOVA, Huynh–Feldt correction to compensate the lack of sphericity. Some variables needed log-, square root- or reciprocal-transformation to reach normality and homogeneity. p values smaller than 0.05 are considered statistically significant. Statistical analyses were performed using SPSS 23.0.0.3 (IBM). Data is visualized as median and interquartile range using GraphPad Prism 7.02. During laboratory analyses, the researcher was blinded to animal characteristics.

Metabolomics data were analysed using MetaboAnalyst 4.0^61^. Missing values were replaced with a small value, being half of the minimum positive value in the original data, and metabolites with > 51% missing values were excluded from analysis. Data were log-transformed to reach normal distribution. Significantly changed metabolites were detected using t-tests, with an adjusted P value (FDR) cutoff of 0.05.

## Supplementary information

Supplementary Information 1.

## Data Availability

The data that support the findings of this study are available from the corresponding author upon reasonable request.
