# Supplementary material for: Mid-gestation low-dose LPS administration results in female-specific excessive weight gain upon a western style diet in mouse offspring
Source: Sci Rep. 2020 Nov 12;10:19618. doi: 10.1038/s41598-020-76501-8 (PMC7665071; doi:10.1038/s41598-020-76501-8)
Supplement: Supplementary file 1 — Supplementary Information 1. [file 41598_2020_76501_MOESM1_ESM.pdf]

Mid-gestation low-dose LPS administration results in female-specific excessive weight gain upon a western style diet in mouse offspring

Dorieke J Dijkstra[1], Rikst Nynke Verkaik-Schakel[1#], Sharon Eskandar[1,2#], Alice Limonciel[3], Violeta Stojanovska[4], Sicco A Scherjon[1], Torsten Plösch[1]*

[1] University of Groningen, University Medical Center Groningen, Department of Obstetrics and Gynaecology, Groningen, The Netherlands
[2] University of Groningen, University Medical Center Groningen, Department of Biomedical Sciences of Cells & Systems, Section Molecular Neurobiology, Groningen, The Netherlands.
[3] Biocrates Life Sciences AG, Austria AT
[4] Helmholtz Centre for Environmental Research, Department of Environmental Immunology, Leipzig, Germany
[#] These authors contributed equally to this work
* Corresponding author

Correspondence address
Torsten Plösch
UMCG, Department of Obstetrics and Gynaecology
Hanzeplein 1, CB22
9713GZ Groningen
The Netherlands
Tel: +31 50 3613149
Fax: +31 50 3611694
t.plosch@umcg.nl

Supplementary figures and tables

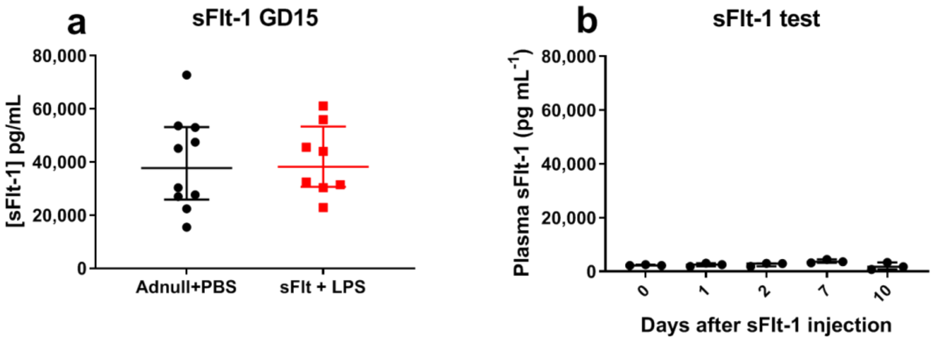

Figure S1. sFlt-1 protein abundance in pregnant and non-pregnant mice after sFlt-1 adenovirus administration. Pregnant mice: sFlt-1 adenovirus was administered at GD8 and protein abundance was determined at GD15, the adenovirus did not induce the protein (**a**). Non-pregnant mice: sFlt-1 adenovirus was administered and protein abundance was determined 1, 2, 7 and 10 day afterwards, the adenovirus did not induce the protein (**b**). Together, these data show the sFlt-1 adenovirus injection had no effect on sFlt-1 protein abundance. LPS: lipopolysaccharides.

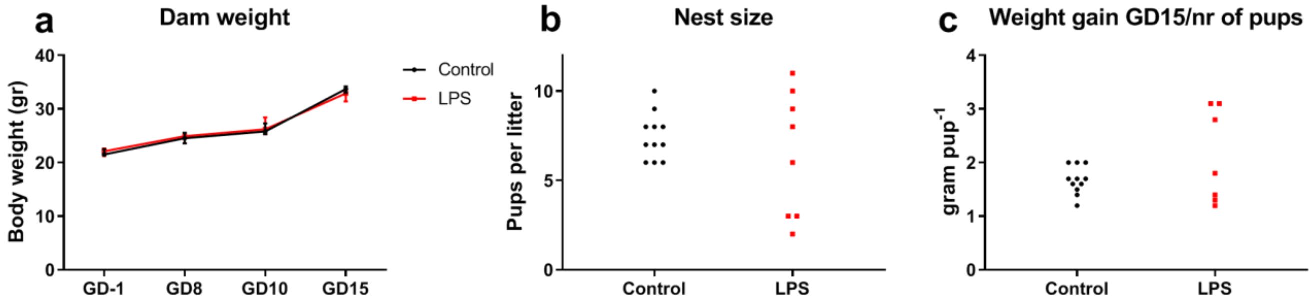

Figure S2. Gestation characteristics of dams with and without LPS treatment at gestational day 10.5. Bodyweight during gestation (**a**), nest size at postnatal day 1 (**b**), and gestational weight gain at GD 15.5 per pup (**c**). Data are shown as median (interquartile range) (a) or as individual measurements (b, c). LPS: lipopolysaccharides.

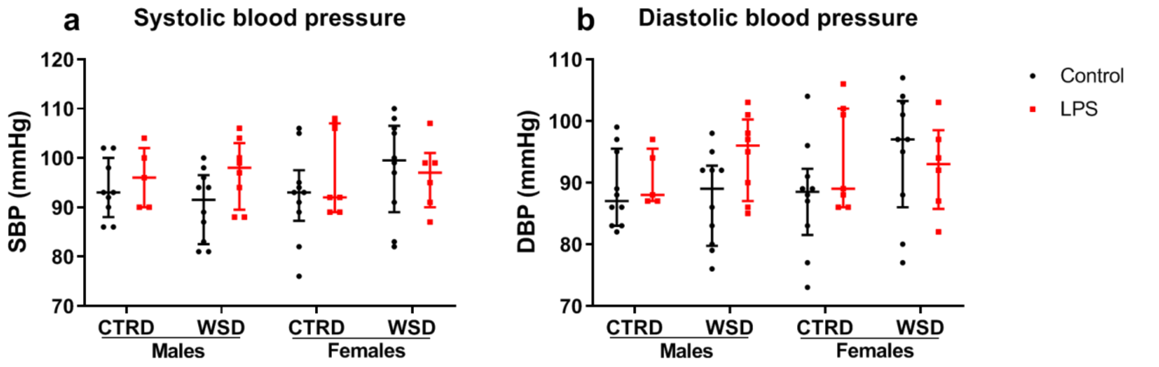

Figure S3. Offspring systolic (**a**) and diastolic (**b**) blood pressure after in utero maternal inflammation exposure combined with a WSD later in life. Analysed using 3-way (repeated measures) ANOVA, data are presented as median (interquartile range). CTRD: control diet; WSD: western-style diet; LPS: lipopolysaccharides.

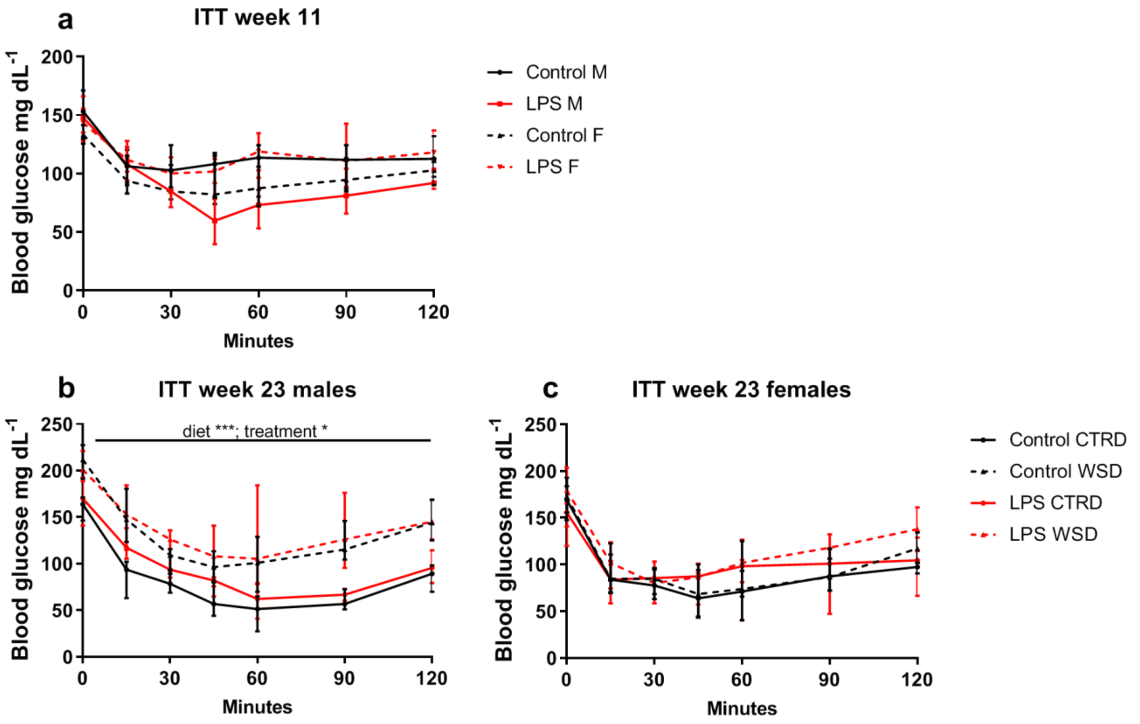

Figure S4. Insulin tolerance test results of offspring exposed to maternal inflammation in utero combined with a WSD later in life. Blood glucose concentrations before and after insulin administration at week 11 (**a**) and 23 (**b** for males, **c** for females). In week 23, males and females were analysed separately because of different insulin doses. Analysed using 3-way repeated measures ANOVA, data are presented as median (interquartile range). *: p<0.05; ***: p<0.001. CTRD: control diet; WSD: western-style diet; LPS: lipopolysaccharides.

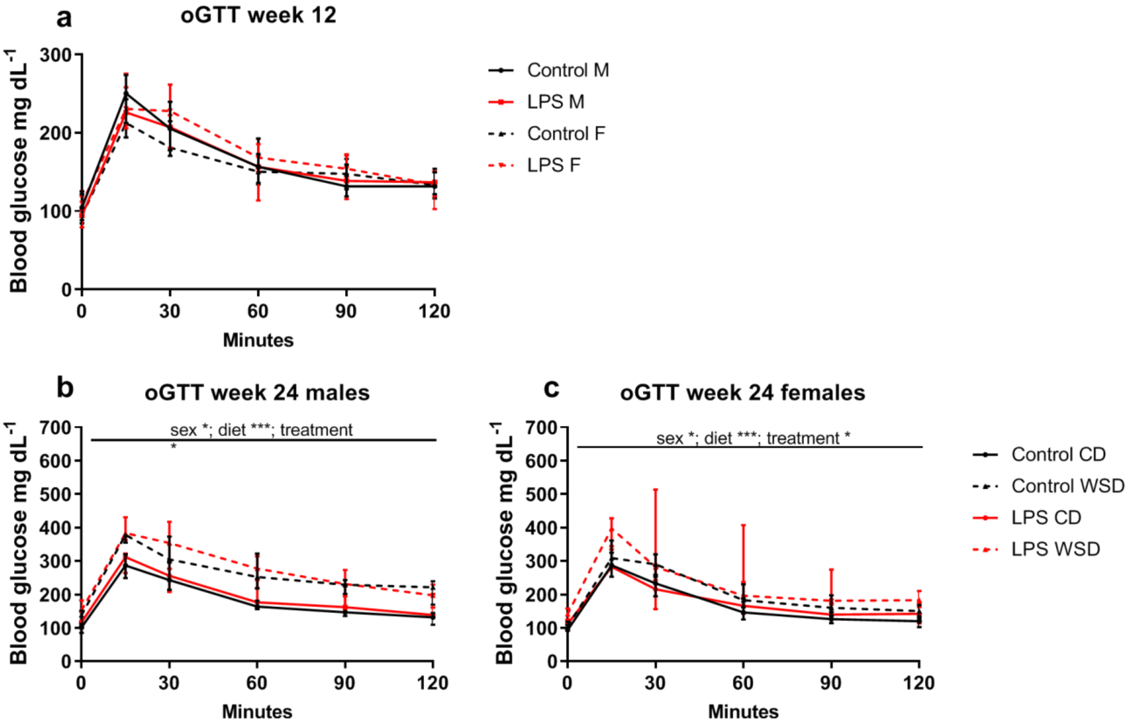

Figure S5. Oral glucose tolerance test results of mice exposed to maternal inflammation in utero combined with a WSD later in life. Blood glucose concentrations before and after administration of a glucose bolus at week 11 (**a**) and 23 (**b** for males, **c** for females). Analysed using 3-way repeated measures ANOVA, data are presented as median (interquartile range). *: $p<0.05$; **: $p<0.01$; ***: $p<0.001$. CTRD: control diet; WSD: western-style diet; LPS: lipopolysaccharides.

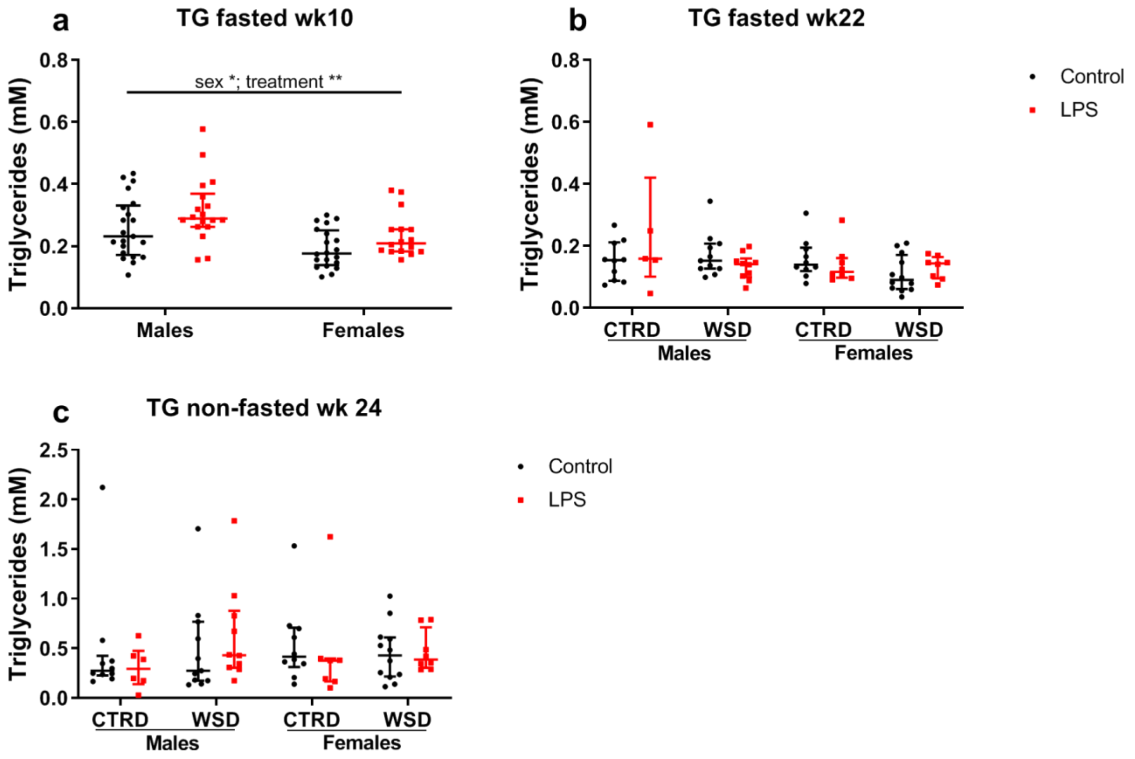

Figure S6. Levels of plasma triglycerides after 6 hours of fasting in week 10 (**a**) and 22 (**b**) and in a non-fasted state in week 24 (**c**). Analysed using 2-way ANOVA (a) and 3-way ANOVA (b, c). Data are presented as median (interquartile range). *: p<0.05; **: p<0.01. CTRD: control diet; WSD: western-style diet; LPS: lipopolysaccharides.

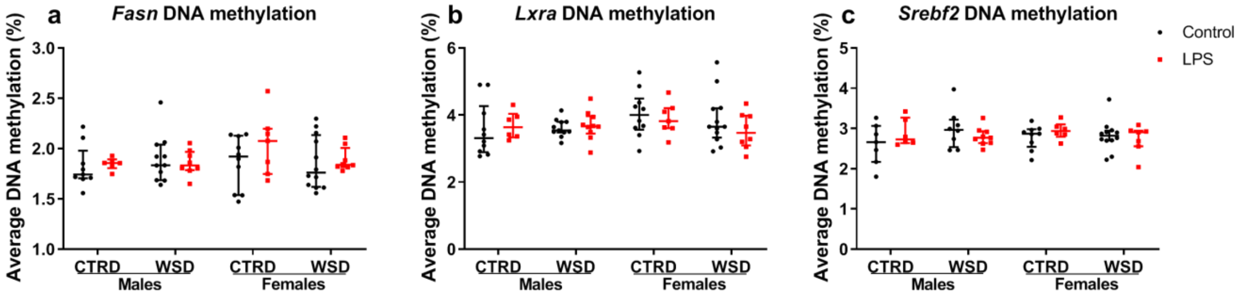

Figure S7. Hepatic DNA methylation of key genes *Fasn*, *Lxra* and *Srebf2* in mice exposed to maternal inflammation in utero combined with a WSD later in life. Average DNA methylation in a selection of the promoter region of *Fasn* (**a**), *Lxra* (**b**), and *Srebf2* (**c**) in the liver. Analysed using 3-way ANOVA, data are presented as median (interquartile range). CTRD: control diet; WSD: western-style diet; LPS: lipopolysaccharides.

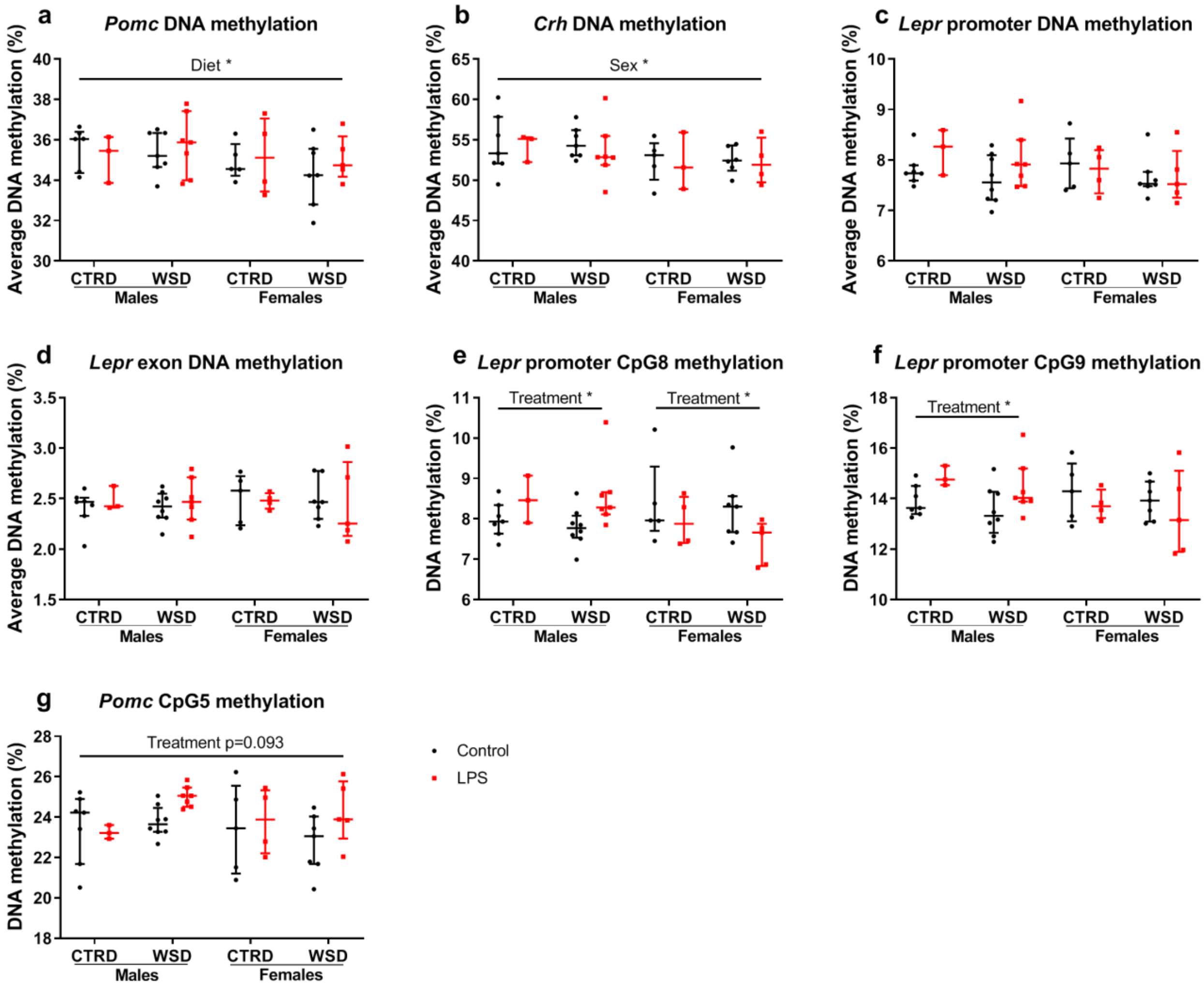

Figure S8. Hypothalamic DNA methylation key genes *Pomc*, *Crh* and *Lepr* in mice exposed to maternal inflammation in utero combined with a WSD later in life. Average DNA methylation of a number of CpG positions in the promoter regions of *Pomc* (**a**), *Crh* (**b**), and *Lepr* (**c**) and in the exon region of *Lepr* (**d**). DNA methylation of specific CpG positions in the *Lepr* promoter (**e, f**) and in the *POMC* promoter (**g**). Analysed using 3-way ANOVA, data are presented as median (interquartile range). *: p<0.05. CTRD: control diet; WSD: western-style diet; LPS: lipopolysaccharides.

Table S1. Tissue weights and relative tissue weights of mice exposed to maternal inflammation in utero combined with a WSD later in life.

	M CTRD Control	M CTRD LPS	M WSD Control	M WSD LPS	F CTRD Control	F CTRD LPS	F WSD Control	F WSD LPS	Significance
**Liver**	1,28 (1,12-1,37)	1,37 (1,33-1,52)	1,93 (1,68-2,49)	1,88 (1,48-2,34)	1,07 (0,94-1,78)	1,07 (1,00-1,13)	1,24 (1,11-1,44)	1,48 (1,30-1,61)	sex***; diet***
**Heart**	0,16 (0,15-0,17)	0,15 (0,14-0,17)	0,16 (0,14-0,18)	0,17 (0,16-0,19)	0,12 (0,12-0,13)	0,12 (0,11-0,12)	0,13 (0,11-0,14)	0,12 (0,12-0,14)	sex***; diet***
**Kidney**	0,16 (0,14-0,17)	0,16 (0,15-0,21)	0,17 (0,15-0,19)	0,17 (0,16-2,05)	0,12 (0,11-0,12)	0,12 (0,11-0,12)	0,12 (0,12-0,14)	0,12 (0,12-0,14)	sex***; diet*; treatment*
**gWAT**	0,56 (0,48-0,71)	0,82 (0,62-1,27)	2,06 (1,83-2,41)	1,83 (1,37-2,36)	0,60 (0,51-0,84)	0,48 (0,46-0,68)	0,81 (0,53-1,09)	1,32 (0,88-1,90)	F WSD*; M CTRD 0.09
**iWAT**	0,12 (0,08-0,14)	0,18 (0,14-0,32)	0,41 (0,32-0,67)	0,43 (0,27-0,61)	0,20 (0,16-0,27)	0,15 (0,10-0,26)	0,23 (0,21-0,31)	0,57 (0,30-0,65)	F WSD*; M CTRD 0.094
**Liver/BW ($x10^{-2}$)**	4,40 (3,89-4,55)	4,51 (4,02-4,78)	5,22 (4,77-6,15)	5,10 (4,38-5,74)	4,35 (4,06-4,64)	4,56 (3,82-4,62)	4,73 (4,34-5,19)	4,71 (4,38-5,05)	diet**
**Heart/BW ($x10^{-3}$)**	5,31 (5,15-5,67)	4,65 (4,41-5,42)	3,89 (3,56-5,20)	4,43 (3,95-5,60)	4,83 (4,61-5,44)	4,82 (4,34-5,18)	4,60 (4,32-5,33)	4,10 (3,82-4,20)	diet*
**Kidney/BW ($x10^{-3}$)**	5,31 (4,88-5,61)	4,86 (4,57-6,96)	4,24 (4,05-5,14)	4,69 (3,88-5,51)	4,70 (4,49-5,06)	4,72 (4,37-5,09)	4,72 (4,35-4,95)	3,90 (3,78-4,83)	diet*
**gWAT/BW ($x10^{-2}$)**	1,87 (1,73-2,44)	2,75 (2,07-3,76)	5,29 (5,08-5,68)	5,05 (4,08-5,70)	2,47 (2,09-3,34)	2,11 (1,93-2,73)	3,23 (2,15-3,88)	4,29 (2,98-5,45)	F WSD *
**iWAT/BW ($x10^{-3}$)**	4,3 (2,8-5,1)	6,0 (4,7-9,4)	11,3 (8,6-16,3)	11,8 (8,5-15,1)	8,1 (6,6-10,7)	6,7 (3,9-10,6)	9,3 (8,5-11,6)	17,6 (10,2-18,9)	F WSD **

Data shown as median (interquartile range). Analysed using 3-way ANOVA with analysis of simple main effects of treatment in case of a treatment-associated interaction. gWAT: gonadal white adipose tissue; iWAT: inguinal white adipose tissue. F: female; M: male; CTRD: control diet; WSD: western-style diet; LPS: lipopolysaccharides.

Table S2. Gene expression in livers of mice exposed to maternal inflammation *in utero* combined with a WSD later in life.

	**M CTRD Control**	**M CTRD LPS**	**M WSD Control**	**M WSD LPS**	**F CTRD Control**	**F CTRD LPS**	**F WSD Control**	**F WSD LPS**	**Significance**
Ppara	0,70 (0,60-0,78)	0,84 (0,76-0,88)	1,14 (0,70-1,40)	1,09 (1,02-1,18)	0,85 (0,75-1,04)	0,77 (0,72-0,86)	1,13 (0,88-1,47)	1,11 (0,91-1,35)	diet ***
Cpt1a	0,62 (0,55-0,87)	0,73 (0,62-0,81)	1,10 (0,86-1,17)	1,00 (0,91-1,05)	0,66 (0,53-0,74)	0,69 (0,67-0,74)	0,87 (0,76-1,14)	0,94 (0,80-1,19)	diet ***
Acaca	0,91 (0,59-1,41)	1,24 (0,86-1,69)	0,90 (0,62-1,45)	0,98 (0,78-1,31)	0,94 (0,69-1,33)	0,80 (0,55-0,83)	0,74 (0,59-1,20)	1,06 (0,64-1,42)	
Scd1	0,61 (0,51-0,67)	0,69 (0,46-0,96)	1,56 (1,32-1,59)	1,26 (1,11-1,66)	0,75 (0,64-0,94)	0,61 (0,42-0,77)	0,93 (0,48-1,28)	1,27 (0,92-1,31)	diet ***
Pparg	0,42 (0,30-0,55)	0,36 (0,32-0,43)	1,24 (0,98-1,53)	0,99 (0,83-1,49)	0,50 (0,38-0,75)	0,58 (0,43-0,72)	0,81 (0,57-1,12)	0,91 (0,79-1,33)	diet in males ***; diet in females ***

Relative expressions are calculated using a standard curve and corrected for the relative expression of *36b4*. Data shown as median (interquartile range). Analysed using 3-way ANOVA with analysis of simple main effects of treatment in case of a treatment-associated interaction. *: $p<0.05$; **: $p<0.01$; ***: $p<0.001$. F: female; M: male; CTRD: control diet; WSD: western-style diet; LPS: lipopolysaccharides;

Table S3. Gene expression in gonadal white adipose tissue of mice exposed to maternal inflammation *in utero* combined with a WSD later in life.

	M CTRD Control	M CTRD LPS	M WSD Control	M WSD LPS	F CTRD Control	F CTRD LPS	F WSD Control	F WSD LPS	Significance
Fasn	0,90 (0,69-1,59)	1,22 (0,85-1,51)	0,32 (0,29-0,39)	0,36 (0,25-0,52)	1,52 (0,47-2,66)	2,29 (1,44-2,79)	0,68 (0,42-1,26)	0,58 (0,51-0,82)	sex **; diet ***
Srebf2	1,18 (1,00-1,31)	1,12 (0,85-1,49)	0,95 (0,76-1,08)	0,87 (0,80-0,98)	1,16 (0,88-1,37)	1,30 (1,14-1,40)	1,08 (0,92-1,25)	0,88 (0,82-1,08)	diet ***
Pparg	1,23 (0,76-1,39)	1,00 (0,91-1,47)	0,61 (0,52-0,76)	0,63 (0,47-0,97)	1,11 (0,96-1,36)	1,46 (1,16-1,72)	1,01 (0,77-1,27)	0,92 (0,87-1,05)	sex ***; diet ***
Zfp423	0,82 (0,73-0,98)	0,96 (0,78-1,18)	0,90 (0,84-0,98)	0,74 (0,63-0,96)	0,97 (0,66-1,36)	1,15 (1,06-1,23)	1,02 (0,86-1,26)	1,21 (1,08-1,37)	sex ***
Scd1	0,86 (0,62-1,06)	0,85 (0,56-0,85)	0,80 (0,53-0,98)	0,67 (0,45-0,98)	1,20 (1,06-1,65)	1,61 (1,11-1,99)	1,01 (0,80-1,50)	0,88 (0,75-0,95)	sex ***; diet **
Chrebf	0,97 (0,80-1,24)	1,02 (0,72-2,03)	0,38 (0,33-0,44)	0,49 (0,31-0,49)	1,16 (1,11-2,33)	2,26 (1,81-2,91)	0,67 (0,52-1,46)	0,58 (0,55-0,90)	sex ***; diet ***
Cdkn2a	0,57 (0,42-1,12)	0,69 (0,46-0,72)	0,99 (0,78-1,56)	0,95 (0,74-1,11)	0,85 (0,69-1,17)	0,98 (0,72-1,57)	1,08 (0,71-1,47)	1,20 (0,66-1,57)	sex *; diet **
11bhsd1	0,92 (0,80-1,06)	1,03 (0,93-1,09)	0,77 (0,61-0,77)	0,83 (0,74-0,84)	1,08 (0,94-1,31)	1,12 (0,95-1,35)	0,96 (0,82-1,17)	1,00 (0,80-1,15)	sex ***; diet ***
Nr3c1	0,72 (0,65-0,85)	0,83 (0,75-0,85)	0,84 (0,66-1,01)	0,88 (0,75-1,07)	0,87 (0,78-1,04)	0,81 (0,65-1,05)	1,00 (0,80-1,16)	1,04 (0,86-1,21)	sex **; diet **

Relative expressions are calculated using a standard curve and corrected for the relative expression of *Actb*. Data shown as median (interquartile range). Analysed using 3-way ANOVA with analysis of simple main effects of treatment in case of a treatment-associated interaction. *: p<0.05; **: p<0.01; ***: p<0.001. F: female; M: male; CTRD: control diet; WSD: western-style diet; LPS: lipopolysaccharides.

Table S4. Composition of diets D12079B and D13100302 (Research Diets, New Brunswick, New Jersey, USA)

	Diet D12079B		Diet D13100302	
	**g %**	**kcal %**	**g %**	**kcal %**
**Protein**	20	17	17	17
**Carbohydrate**	50	43	71	73
**Fat**	21	40	4	10
**Total**		100		100
**kcal/g**	4,68		3,91	
**Ingredient**	**g**	**kcal**	**g**	**kcal**
**Casein, 80 Mesh**	195	780	195	780
**DL-Methionine**	3	12	3	12
**Corn starch**	50	200	695,4	2782
**Maltodextrin 10**	100	400	150	600
**Sucrose**	341	1364	0	0
**Cellulose,BW200**	50	0	50	0
**Milk fat, anhydrous**	200	1800	0	0
**Corn oil**	10	90	52,5	473
**Ethoxyquin**	0,04	0	0,04	0
**Mineral mix S10001**	35	0	35	0
**Calcium carbonate**	4	0	4	0
**Vitamin Mix V10001**	10	40	10	40
**Choline bitartrate**	2	0	2	0
**Cholesterol**	1,5	0	0	0
**Total**	**1001.54**	**4686**	**1197,04**	**4686**

Table S5. Primers and probes used for qPCR in this study.

Liver	**Forward**	**Reverse**	**Probe**
Lxra	TGC CTG ATG TTT CTC CTG ATT CT	CCT CCC TGG TCT CCT GCA T	TTG AGG TTC TGT CTT CCA CAA CTC CGT TG
Srebf2	CTG CAG CCT CAA GTG CAA AG	CAG TGT GCC ATT GGC TGT CT	CCA TCC AGC AGC AGG TGC AGA CG
Fasn	GGC ATC ATT GGG CAC TCC TT	GCT GCA AGC ACA GCC TCT CT	CCA TCT GCA TAG CCA CAG GCA ACC TC
Elovl6	ACA CGT AGC GAC TCC GAA GAT	AGC GCA GAA AAC AGG AAA GAC T	TTT CCT GCA TCC ATT GGA TGG CTT C
Chrebf	GAT GGT GCG AAC AGC TCT TCT	CTG GGC TGT GTC ATG GTG AA	CCA GGC TCC TCC TCG GAG CCC
Ppara	TAT TCG GCT GAA GCT GGT GTA C	CTG GCA TTT GTT CCG GTT CT	CTG AAT CTT GCA GCT CCG ATC ACA CTT G
Cpt1a	CTC AGT GGG AGC GAC TCT TCA	GGC CTC TGT GGT ACA CGA CAA	CCT GGG GAG GAG ACA GAC ACC ATC CAA C
Srebf1c	GGA GCC ATG GAT TGC ACA TT	CCT GTC TCA CCC CCA GCA TA	CAG CTC ATC AAC AAC CAA GAC AGT GAC TTC C
Acaca	CCA TCC AAA CAG AGG GAA CAT C	CTA CAT GAG TCA TGC CAT AGT GGT T	ACG CTA AAC AGA ATG TCC TTT GCC TCC AAC
Scd1	ATG CTC CAA GAG ATC TCC AGT TCT	CTT CAC CTT CTC TCG TTC ATT TCC	CCA CCA CCA CCA TCA CTG CAC CTC
Pparg	CAC AAT GCC ATC AGG TTT GG	GCT GGT CGA TAT CAC TGG AGA TC	CCA ACA GCT TCT CCT TCT CGG CCT G
36b4	GCT TCA TTG TGG GAG CAG ACA	CAT GGT GTT CTT GCC CAT CAG	TCC AAG CAG ATG CAG CAG ATC CGC
Cidea	GGC CGT GTT AAG GAA TCT GC	GTA TGT GCC CGC ATA GAC CA	
Cidec	CCT ATG ACC TGC ACT GCT ACA AG	CAT GTA GCT GGA GGT GCC AAG	
gWAT			
Leptin	AAG ACC ATT GTC ACC AGG ATC AA	GGT CCA TCT TGG ACA AAC TCA GA	CAC ACA CGC AGT CGG TAT CCG CC
Zfp423	GCA TGG GCG GTA CCT TCA	GCA CGG CAA AGA TAT GCT GTT	TCT GCT TCA CAG TCT TCG TCC AGG CC
Cdkn2a	AGA CCG ACG GGC ATA GCT T	TAG CTC TGC TCT TGG GAT TGG	TCA AGC ACG CCC AGG GCC CT
11bhsd1	ATG AAG AGT TCA GAC CAG AAA TGC T	ACA GAT GAT ATG CCA TTT CTC TTC C	CTT TGC TGG CCC CAG TGA CAA TCA CT
Nr3c1	GGA TAT TCA AGC CCT GGA ATG A	ACG TCA GCA CCC CAT AAT GG	ACC ACC TCC CAA ACT CTG CCT GGT GT
Actb	AGC CAT GTA CGT AGC CAT CCA	TCT CCG GAG TCC ATC ACA ATG	TGT CCC TGT ATG CCT CTG GTC GTA CCA C
Fasn, Srebf2, Pparg, Scd1, Chrebf **and** ***Lxra:*** **primers sequences see Liver**			
Hypothalamus			
Npy	TCT TAA TGA AGG AAA GCA CAG AAA AC	TTT CAT TTC CCA TCA CCA CAT G	
Agrp	GCT AGA TCC ACA GAA CCG CGA G	ATT GAA GAA GCG GCA GTA GCA CGT G	

Socs3	GGA CCA AGA ACC TAC GCA TCC A	CAC CAG CTT GAG TAC ACA GTC G
Lepr	GAA GCA CTG TGC AGC TGA GG	GCA GTT TTT GGG CTC AGA CG
Pomc	TGC TTC AGA CCT CCA TAG ATG TGT	GGA TGC AAG CCA GCA GGT T
Cartpt	ACG AGA AGA AGT ACG GCC AAG T	CCG ATC CTG GCC CCT TT
Mc3r	CTT GGC TGT TGC TTC TTT TGC	TTG CCG ACA GGG TCA TTT G
Mc4r	TAG CCT GGC TGT GGC AGA T	CGA TGG TTT CCG ACC CAT T
Crh	CCT CAG CCG GTT CTG ATC C	GCG GAA AAA GTT AGC CGC AG
Fkbp5	GCG GCG ACA GGT CTT CTA CT	CAC CCT GCT CAG TCA TGG TG
Cnr1	GTG CTG TTG CTG TTC ATT GTG	CTT GCC ATC TTC TGA GGT GTG
Hprt	TGA CAC TGG CAA AAC AAT GCA	GGT CCT TTT CAC CAG CAA GCT
β-actin	AGC CAT GTA CGT AGC CAT CCA	TCT CCG GAG TCC ATC ACA ATG
Cortex		
Gapdh	TGG CAA AGT GGA GAT TGT TGC C	AAG ATG GTG ATG GGC TTC CCG
Apoe	TGT GGG CCG TGC TGT TGG TC	GCC TGC TCC CAG GGT TGG TTG
Cd68	CCA CAG GCA GCA CAG TGG ACA	GCA GAA GCT TTG GCC CAA GGG A
Bdnf	CTC TGG AGA GCG TGA ATG GG	CGT GCT CAA AAG TGT CAG CC
Aqp4	TCA GCA TCG CTA AGT CCG TC	TCC ATG AAC CGT GGT GAC TC
Tyrobp	GAT TGC CCT GGC TGT GTA CT	TGT TGT TTC CGG GTC CCT TC
H2aa	TAC CAA TGA GGC TCC TCA AGC	CGT CTG CGA CTG ACT TGC TA
Cxcl10	GGG TCT GAG TGG GAC TCA AGG GAT	GGC CCT CAT TCT CAC TGG CCC
Vista	AAC AAC GGT TCT ACG GGT CC	CGT GAT GCT GTC ACT GTC CT
Slc1a2	AAG CCT TAC AGC CAC CCT G	CTC ATT CTA TCC AGC AGC CAG TC
Ctsd	TCT ACC TGA AGC TAG GAG GCA	CAC CCT GCG ATA CCT TGA GT
Cx3cr1	CTT GCC TCT GGT GGA GTC TG	GTG AGG TCC TGA GCA GAT GG
Fgfr3	GCG ACA GGT GTC CTT GGA AT	GCC AGA ACA GGA CCT TCT CC
Tmem119	CTT CAC CCA GAG CTG GTT CC	GTG ACA CAG AGT AGG CCA CC
Axl	TGA AGC CAC CTT GAA CAG TC	GCC AAA TTC TCC TTC TCC CA
Gfap	GGA GGT GGA GAG GGA CAA C	GTT TCA TCT TGG AGC TTC TGC
P2ry12	CAA CTC ACC TTC ACC GGC A	GCC TTG AGT GTT TCT GTA GGG T

Table S6. Primers used for amplification and pyrosequencing of bisulfite-treated DNA.

	Forward 5'-3'	Reverse 5'-3'	Sequencing primer	Sequence to analyze
Lxra	TTTTGGTAGAGGGTAGGGGATAG	Biotin-ACCCAAAACTCTTAACTTCATTAAAC	GGTAGGGGATAGGGT	GGYGTYGGTTAAGAGGGTGGTYGGTYGGGGAGGAGTTAGTTT
Fasn	TTTTAAGGTGGTTATAGAGGG	Biotin-ACAACAACCACAAAACACTT	GTTATAGAGGGTGGGA	GTTYGAGAAAGTTGGGTTAYGATGATYGGTAGTAATTTYGTTTGAGGYGTTTTTYGTTAGGGTTAAYGATYGYGTTTGYGYGGGGGTTYGYGAAG
Srebf2	GGTTAATGTAGGTTTGGTTTTATTGAT	Biotin-ATAACACCCCACAACCCCCACCTAATA	GTAGGTTTGGTTTTATTGATAATA	AATTTGGGGGTGYGGAGGTTYGGGGYGGGGTTGTAGTGGGYGYGGTTYGGGGYGGGGGAA
***Lepr* promoter**	Biotin-GTTGTAAAGGTTAGAGAGGATAGAAT	AACTAAAAAAATATCCCACCTATATCC	ACACTAACCACTCAAAT	AACTACRCRACRAAAACRCTACRCRCRTAACCRAAAAACTTAAACRAAACTTTAAAAACTAATTCTATCC
***Lepr* exon**			ACTCTATACTACTAACTCAAAA	AATACCRACCACTCRTACRCRAACRCRACRCTACRAAACACTAACCACTCAAATAACTAC
***Pomc* promoter seq 1**	Biotin-TAGGGTTGGGTGGGTGAG	CACAAAAACCCTAAACCTCTATCCAATTCT	CTTTCAAACAAATATACCTT	ACRCTCAACCAAAACCRAAAAACCCCCCTCCCRAAACCCRCCRCCCCCCTTCRCTACAAAAACRCTACCAAAAAAATCAC
***Pomc* promoter seq 2**			ATTAAATTCTTCCTAACCAC	CAAACRCCAAATATACRCTTCAACRAATCTATACTAACRCCAACCTCCRCRCTTTCCAAACAAATATACCT TAC
Crh	TGGTAGGGTTTTATTATTTATGTAGGAGTA	Biotin-TACCTTTTCCCTTTCTCTTCAATCTCT	ATGTAGGAGTAGAGGTAGTA	YGTAATYGAGTTGTTAAGAGAGYGTTAGTTTATTAGGTAAATGTTGYGTGTTTTTTGAAGAGGGTYGATATTATAAAATTTTATTTTAGGT
